# Ubiquitination-Related Molecular Subtypes and a Novel Prognostic Index for Bladder Cancer Patients

**DOI:** 10.3389/pore.2021.1609941

**Published:** 2021-10-29

**Authors:** Hai Cai, Hang Chen, Qi Huang, Jun-Ming Zhu, Zhi-Bin Ke, Yun-Zhi Lin, Qing-Shui Zheng, Yong Wei, Ning Xu, Xue-Yi Xue

**Affiliations:** ^1^ Department of Urology, Urology Research Institute, The First Affiliated Hospital, Fujian Medical University, Fuzhou, China; ^2^ Fujian Key Laboratory of Precision Medicine for Cancer, The First Affiliated Hospital, Fujian Medical University, Fuzhou, China

**Keywords:** prognosis, bladder cancer, ubiquitination, molecular subtypes, prognostic index

## Abstract

**Objective:** To develop and validate ubiquitination-related molecular subtypes and a novel prognostic index using ubiquitination-related genes (URGs) for patients with bladder cancer (BCa).

**Materials and Methods:** We downloaded the clinical data and transcriptome data of BCa from TCGA and GEO database. Consensus clustering analysis was conducted to identify ubiquitination-related molecular subtypes for BCa. Besides, we performed univariate and multivariate Cox regression analysis to develop a novel prognostic URGs-related index for BCa. We conducted internal and external verification in TCGA cohort and GEO cohort, respectively. Furthermore, the associations of ubiquitination-related molecular subtypes and prognostic index with tumor immune environment were also investigated.

**Results:** A total of four ubiquitination-related molecular subtypes of BCa were finally identified. These four molecular subtypes had significantly different clinical characteristics, prognosis, PD-L1 expression level and tumor microenvironment. Besides, we developed a novel prognostic index using six URGs (including HLA-A, TMEM129, UBE2D1, UBE2N, UBE2T and USP5). The difference in OS between high and low-risk group was statistically significant in training cohort, testing cohort, and validating cohort. The area under ROC curve (AUC) for OS prediction was 0.736, 0.723, and 0.683 in training cohort, testing cohort, and validating cohort, respectively. Multivariate survival analysis showed that this index was an independent predictor for OS. This prognostic index was especially suitable for subtype 1 and 3, older, male, high grade, AJCC stage III-IV, stage N0, stage T3-4 BCa patients.

**Conclusions:** This study identified a total of four ubiquitination-related molecular subtypes with significantly different tumor microenvironment, prognosis, clinical characteristics and PD-L1 expression level. Besides, a novel ubiquitination-related prognostic index for BCa patients was developed and successfully verified, which performed well in predicting prognosis of BCa.

## Introduction

The new cases of bladder cancer (BCa) increase by more than 500,000 per year and the deaths caused by BCa increase by approximately 200,000 per year [1,2]. The treatment outcomes are diverse for different BCa patients, especially muscle-invasive BCa (MIBC) [3]. Previously, there were five major subtyping classification systems, including LUND, UROMOL, the University of North Carolina (UNC), The Cancer Genome Atlas (TCGA), and the MD Anderson Cancer Center (MDACC). These five subtyping classification systems not only have evolved independently, but also each taxonomy is different among each other in nomenclature [4–8]. Until now, there is no consistent risk stratification for BCa [9].

Ubiquitination is one of proteins post-translational modification types, and proteins ubiquitination might alter proteins localization, lead to degradation via proteasome, affect proteins activity and interactions [10,11]. Recent studies demonstrated that ubiquitination played vital roles in cancer-related pathways, and that ubiquitinated protein accumulation might be a novel method for treating cancer [12,13]. Akinori Sato et al. [12], reported that ixazomib and ritonavir could suppress the tumorigenesis of BCa by inducing the accumulation of ubiquitinated protein. The development of targetable molecular subtypes and risk stratification tools using ubiquitination-related genes (URGs) is promising. However, as far as we know, there is no previous study exploring the correlations of ubiquitination with prognosis evaluation and molecular subtypes of BCa.

In our study, consensus clustering analysis was used to identify ubiquitination related molecular subtypes. Besides, we developed a novel prognostic index based on URGs by multivariate Cox regression analysis. Next, we conducted internal and external verification, respectively. Finally, we also explored the correlations of the URGs-based prognostic index with molecular subtypes, clinical features, immune function and immune infiltrating cells.

## Materials and Methods

### Data Acquisition

The transcriptome and clinical data of BCa were obtained from TCGA database (https://portal.gdc.cancer.gov), including 414 BCa cases and 19 normal cases [14]. The clinical features of BCa patients included age, gender, grade, T stage, N stage, M stage, and AJCC stage. A total of 412 BCa cases have clinical data; however, there was only a total of 396 BCa cases with unabridged transcriptome and clinical data simultaneously. Besides, a total of 165 BCa patients with complete mRNA expression profile and clinical data were downloaded from GSE13507 dataset in GEO database (https://www.ncbi.nlm.nih.gov). All these data were processed using Perl language and R language. The clinicopathologic feature of TCGA cohort and GEO cohort was showed in Table 1. We used the Ensemble database (http://asia.ensembl.org/signature.html) to convert Ensemble IDs into gene symbols.

**TABLE 1:** clinicopathologic data of TCGA cohort and GEO cohort.

Variables	TCGA cohort	GEO cohort
Age	68.09 ± 10.57	65.18 ± 11.93
Gender		
Male	304 (73.7%)	135 (81.8%)
Female	108 (26.3%)	30 (18.2%)
Grade		
High	388 (94.1%)	60 (36.3%)
Low	21 (5.0%)	105 (63.7%)
Unknown	3 (0.9)	0 (0%)
T stage		
T0	1 (0.2%)	24 (14.5%)
T1	3 (0.7%)	80 (48.4%)
T2	120 (29.1%)	31 (18.7%)
T3	196 (47.5%)	19 (11.5%)
T4	59 (14.3)	11 (6.9%)
TX	1 (0.2%	0 (0%)
Unknown	32 (8%)	0 (0%)
N stage		
N0	239 (58.0%)	149 (90.3%)
N1	47 (11.4%)	8 (4.8%)
N2	76 (18.4%)	6 (3.6%)
N3	8 (1.9%)	1 (0.65%)
NX	36 (8.7%)	1 (0.65%)
Unknown	6 (1.6%)	0 (0%)
M stage		
M0	196 (47.5%)	158 (95.7%)
M1	11 (2.6%)	7 (4.3%
MX	202 (49.0%)	0 (0%)
Unknown	3 (0.9%)	0 (0%)
Systemic chemotherapy		
Yes	-	27 (16.4%)
No	-	138 (83.6%)
Unknown	-	0 (0%)
Recurrence		
Yes	-	36 (21.9%)
No	-	67 (40.6%)
Unknown	-	62 (37.5%)
Progression		
Yes	-	31 (18.8%)
No	-	134 (81.2%)
Unknown	-	
Intravesical therapy		
Yes	-	56 (33.9%)
No	-	47 (28.6%)
Unknown	-	62 (37.5%)
Cancer-specific survival		
Yes	-	133 (80.6%)
No	-	32 (19.4%)
Unknown	-	0 (0%)
Overall survival		
Yes	232 (56.3%)	96 (58.1%)
No	180 (43.7%)	69 (41.9%)
Unknown	0 (0%)	0 (0%)

The Molecular Signatures Database (MSigDB) (https://www.gsea-msigdb.org/gsea/msigdb) is a collection of annotated gene sets using GSEA software. We extracted 79 URGs from REACTOME PROTEIN UBIQUITINATION gene set (C2: curated gene sets; systematic name: M27742) from MSigDB database. All these URGs were related to protein ubiquitination. The detailed list of these 79 URGs were presented in Supplementary Table S1.

### Identification of Ubiquitination-Related Molecular Subtypes Using Consensus Clustering Analysis

Firstly, the expression matrix of 79 URGs was extracted from BCa transcriptome and merged with overall survival (OS) time using Perl language. There was a total of 396 BCa cases with complete OS data and mRNA expression data in TCGA database. We then performed univariable Cox regression analysis to screen prognostic URGs associated with OS. The cut-off *p* value was set as 0.05. Next, consensus clustering analysis was performed for identifying ubiquitination-related molecular subtypes of BCa patients using R package “ConsensusClusterPlus”. The association between ubiquitination-related molecular subtypes and OS was explored using R package “survival” and “survminer”. The associations between ubiquitination-related subtypes and clinicopathologic characteristics (including gender, grade, T stage, N stage, stage, and age) were presented by utilizing R package “pheatmap”.

### Development and Validation of a Novel Ubiquitination-Based Prognostic Index

First of all, we divided randomly all BCa patients in TCGA database into training cohort and testing cohort (internal verification). All cases in GSE13507 dataset were used as validating cohort (external verification). Then, univariate and multivariate Cox regression analysis were conducted to establish a ubiquitination-based prognostic index for predicting OS of BCa using training cohort.

Next, all cases in training cohort, testing cohort and validating cohort were categorized into low-risk group and high-risk group based on the median risk score. We then performed survival analysis and the time-dependent receiver operating characteristic (ROC) curve to explore the performance of this ubiquitination-based prognostic index in training cohort, testing cohort and validating cohort. The expression heatmap, the distribution of risk score and survival time of training cohort, testing cohort and validating cohort were presented using “pheatmap” R package. Moreover, we performed univariate and multivariate independent prognostic analysis to demonstrate whether this ubiquitination-based prognostic index was an independent predictor of OS in BCa patients.

### Exploration of Immune Cells Infiltration, Tumor Microenvironment, PD-L1 Expression Level

The ESTIMATE algorithm was utilized to evaluate tumor microenvironment (TME) scores while the CIBERSORT method was used to calculate the scores of 22 types of immune infiltrating cells [15,16]. Then, we investigated the associations of ubiquitination-related molecular subtypes with BCa tumor microenvironment and immune infiltrating cells. In addition, the association of the ubiquitination-related molecular subtypes with PD-L1 gene expression level [17] was also explored.

Single sample gene set enrichment analysis (ssGSEA) was conducted to calculate the infiltrating score of 16 types of immune infiltrating cell and the activity of 13 types of immune-related function in TCGA cohort using R package “gsva”. We then investigated the associations of the ubiquitination-based prognostic index with immune infiltrating cells and immune function activity. Besides, the associations of ubiquitination-based prognostic index with TME scores were explored.

### Validation of Six Risk URGs and Functional Enrichment

UALCAN database (http://ualcan.path.uab.edu/) is a portal for facilitating tumor subgroup gene expression and survival analyses. The mRNA expression levels of risk URGs between normal and tumor tissues were demonstrated using UALCAN database. The prognostic value of risk URGs was validated in GEO cohort using univariate Cox regression analysis. Kyoto Encyclopedia of Genes and Genomes (KEGG) functional enrichment for high-risk group and low-risk group was performed using Gene Set Enrichment Analysis (GSEA) method in the whole TCGA cohort.

### Statistical Methods

Statistical analysis was performed utilizing R programming language. Univariate and multivariate Cox regression analysis were performed to establish a ubiquitination-related prognostic index for predicting OS of BCa. Univariate and multivariate independent prognostic analysis were used to demonstrate whether this ubiquitination-related prognostic index was an independent predictor of OS. Survival analysis and the time-dependent receiver operating characteristic (ROC) curve were performed to explore the performance of ubiquitination-related prognostic index. Statistical significance was considered at the level of *p* value <0.05.

## Results

### Identification of Four Ubiquitination-Related Molecular Subtypes

**FIGURE 1:**
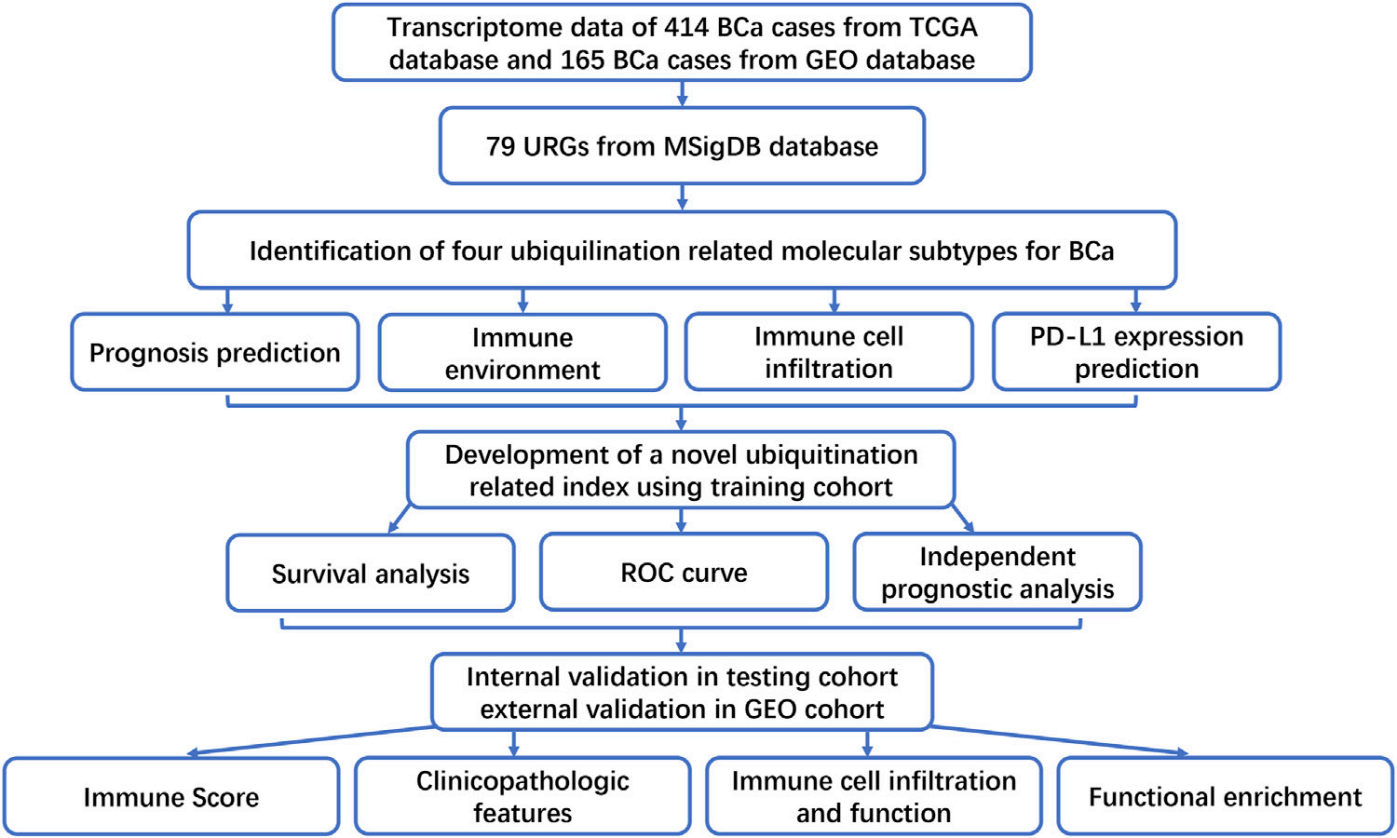
The flowchart of this study.

**FIGURE 2:**
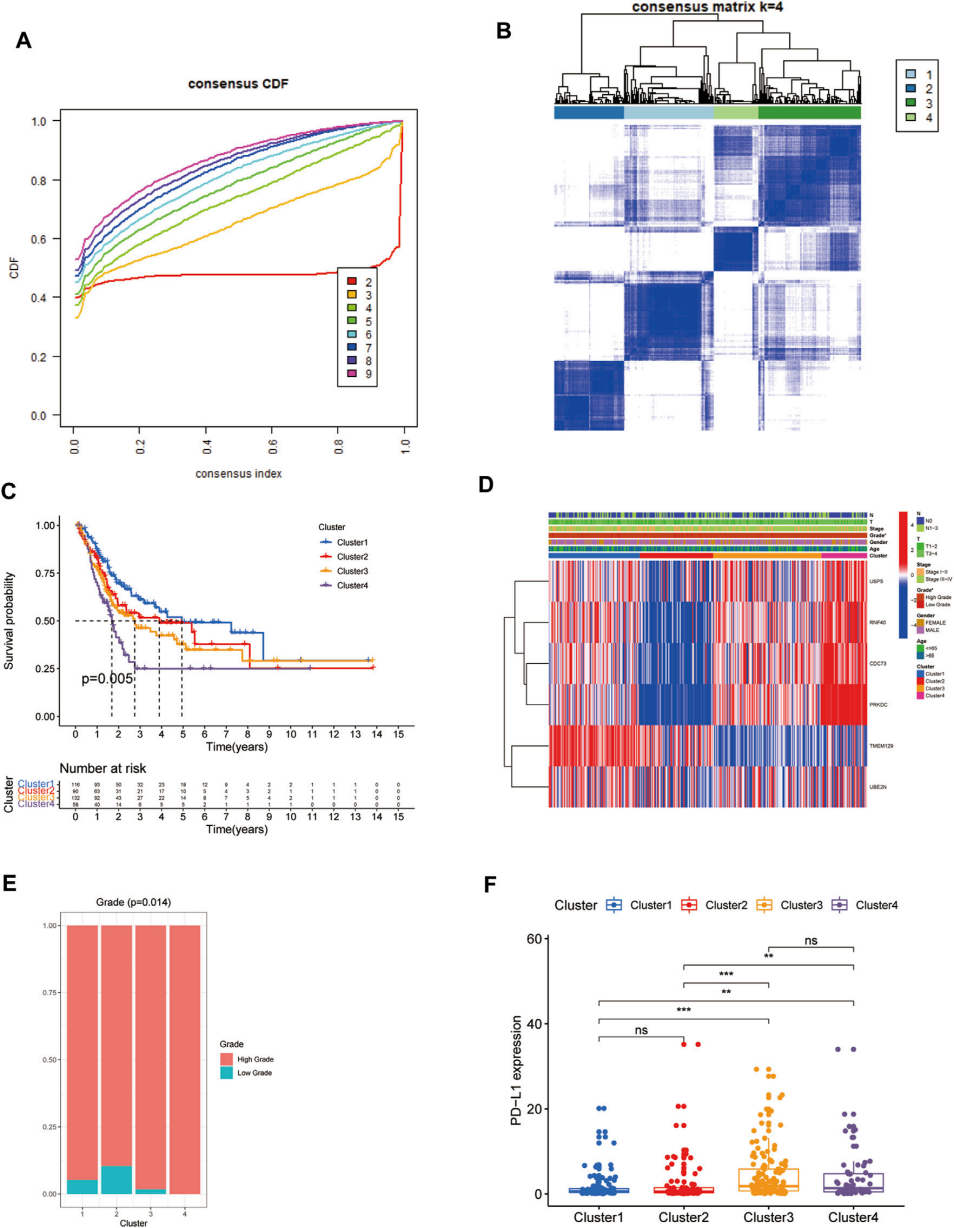
Identification of four ubiquitination-related molecular subtypes of BCa using consensus clustering analysis **(A**, **B)**. Comparison of overall survival among these four subtypes **(C)**. The correlation heatmap between this ubiquitination-related molecular subtypes and clinicopathologic features **(D)**. The difference of grade **(E)** and the expression level of PD-L1 among these four subtypes **(F)**. *p < 0.05; **p < 0.01; ***p < 0.001.

The flowchart of this study was presented in Figure 1. Firstly, the expression matrix of 79 URGs was extracted from TCGA database and merged with OS status and time. We then conducted univariable Cox regression analysis in the whole TCGA cohort to screen URGs associated with OS. The results showed that there was a total of six URGs associated with OS, including CDC73, PRKDC, RNF40, TMEM129, UBE2N and USP5. Next, the expression matrix of these six URGs was used to conduct consensus clustering analysis to identify ubiquitination-related molecular subtypes of BCa. Finally, a total of four ubiquitination-related molecular subtypes of BCa were identified, including 116 cases of subtype 1, 90 cases of subtype 2, 132 cases of subtype 3, 58 cases of subtype 4 (Figures 2A,B). The difference of OS among these ubiquitination-related molecular subtypes was statistically significant (*p* = 0.005, Figure 2C). As indicated by heatmap and histogram, these four molecular subtypes have significantly different grade (*p* < 0.05, Figures 2D,E **).**

### Associations of these Subtypes with PD-L1 Expression and TME

We explored the discrepant expression level of PD-L1 in TCGA database among these four molecular subtypes. The results showed that the expression level of PD-L1 in subtype 3 and subtype 4 was significantly increased compared with that in subtype 1 and subtype 2. However, the PD-L1 expression level was similar between subtype 1 and subtype 2, between subtype 3 and subtype 4, respectively. (Figure 2F).

**FIGURE 3:**
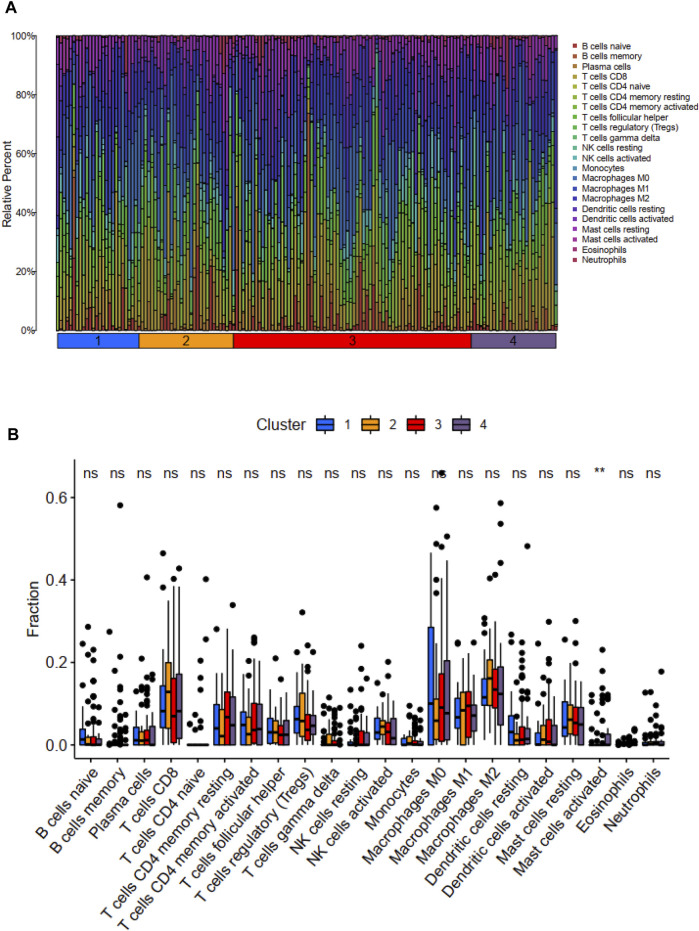
Distribution of 22 types of immune cells among these four molecular subtypes **(A)**. Relationship between these ubiquitination-related molecular subtypes and immune cells infiltration **(B)**. *p < 0.05; **p < 0.01; ***p < 0.001.

**FIGURE 4:**
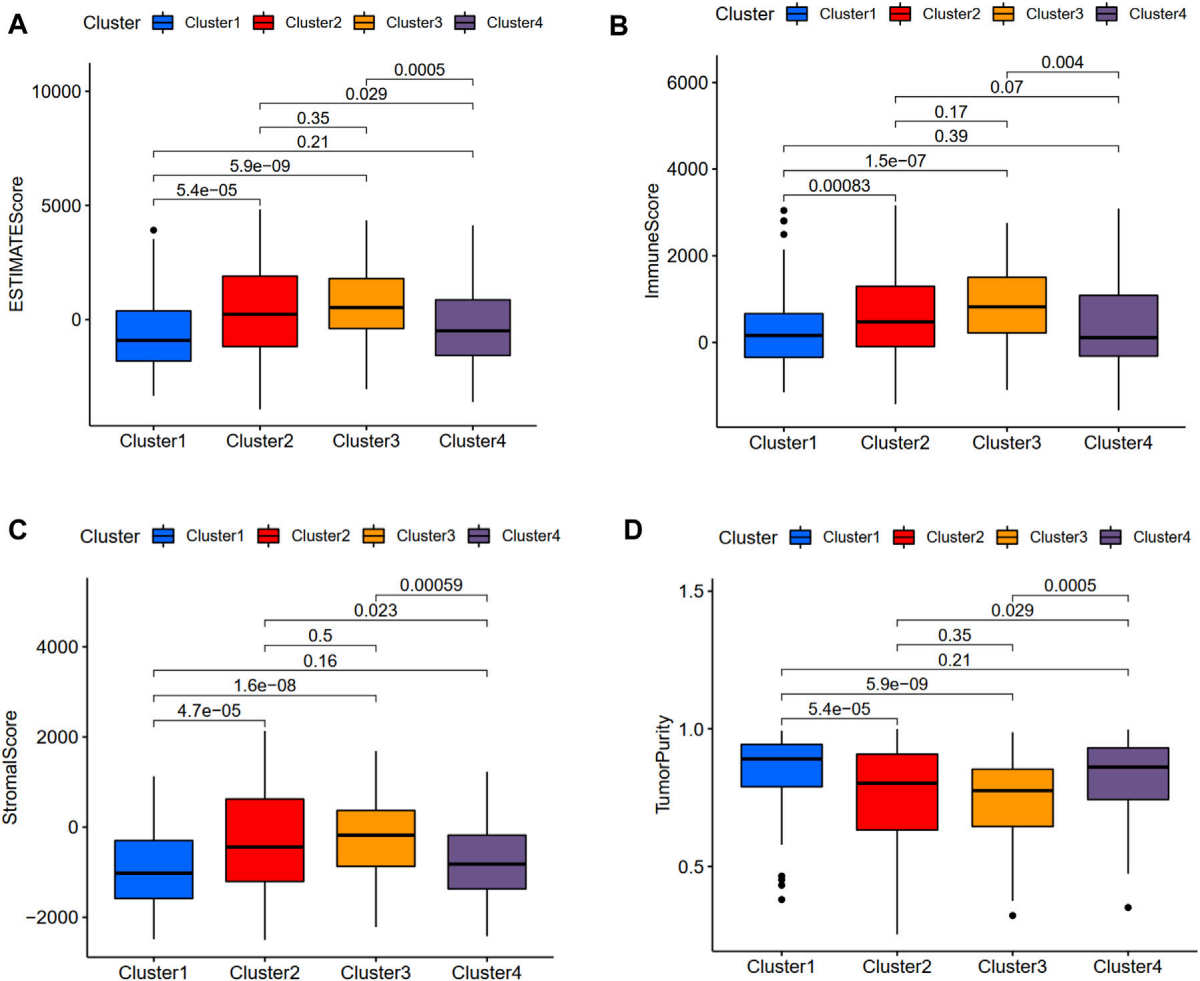
Relationship between the ubiquitination-related molecular subtypes and tumor microenvironment. ESTIMATE score **(A)**. Immune score **(B)**. Stromal score **(C)**. Tumor purity **(D)**.

We used the CIBERSORT method to calculate the scores of 22 types of immune infiltrating cells and the ESTIMATE algorithm to evaluate TME. According to the CIBERSORT method, the activated mast cell was the exclusively discrepant immune infiltrating cell among these four ubiquitination-related subtypes (Figure 3). The ESTIMATE scores, immune scores, stromal scores in subtype 2 and subtype 3 were significantly higher in comparison with that in subtype 1 and subtype 4; the tumor purity scores in subtype 1 and subtype 4 were significantly increased compared with that in subtype 2 and subtype 3. However, the ESTIMATE scores, immune scores, stromal scores and tumor purity scores were similar between subtype 1 and subtype 4, between subtype 2 and subtype 3, respectively (Figure 4).

### Development and Validation of a Novel Ubiquitination-Based Prognostic Index

There was a total of 200 cases in training cohort, 196 cases in testing cohort and 165 cases in validating cohort. Firstly, univariate and multivariate Cox regression analyses were conducted to develop a novel ubiquitination-based index utilizing six URGs (including HLA-A, TMEM129, UBE2D1, UBE2N, UBE2T and USP5) for prognosis prediction of BCa using training cohort. The calculation formula of risk score is shown as follows: Risk score= (−0.0010853) * HLA-A + (−0.03553995) * TMEM129 + (−0.08324461) * UBE2D1 + (−0.07059857) * UBE2N + (0.01191996) * UBE2T + (0.02752775) * USP5 (Table 2).

**TABLE 2:** Univariate and multivariate Cox regression analysis in training cohort to developing ubiquitination-based prognostic index for bladder cancer.

Id	Univariate				Multivariate				
	HR	Low 95% CI	High 95% CI	*p* Value	coef	HR	Low 95% CI	High 95% CI	*p* Value
HLA-A	0.99915356	0.99837245	0.99993528	0.03382399	−0.0010853	0.99891529	0.99813931	0.99969187	0.00619601
PRKDC	1.04297385	1.0110097	1.07594858	0.00806277	-	-	-	-	-
RNF40	1.06154033	1.00293457	1.12357068	0.03929409	-	-	-	-	-
TMEM129	0.96400073	0.93064592	0.998551	0.04128326	−0.03553995	0.96508418	0.9310928	1.00031648	0.05205632
UBE2D1	0.86972657	0.77892987	0.97110706	0.01309643	−0.08324461	0.92012605	0.82062204	1.03169534	0.15398551
UBE2N	0.9451822	0.89434955	0.99890404	0.04562545	−0.07059857	0.93183588	0.87441572	0.99302664	0.02958425
UBE2T	1.01360811	1.0013501	1.02601618	0.02945819	0.01191996	1.01199129	0.99783483	1.02634858	0.09723643
USP5	1.03053798	1.00884729	1.05269502	0.00557936	0.02752775	1.02791013	1.00665979	1.04960907	0.00980211

**FIGURE 5:**
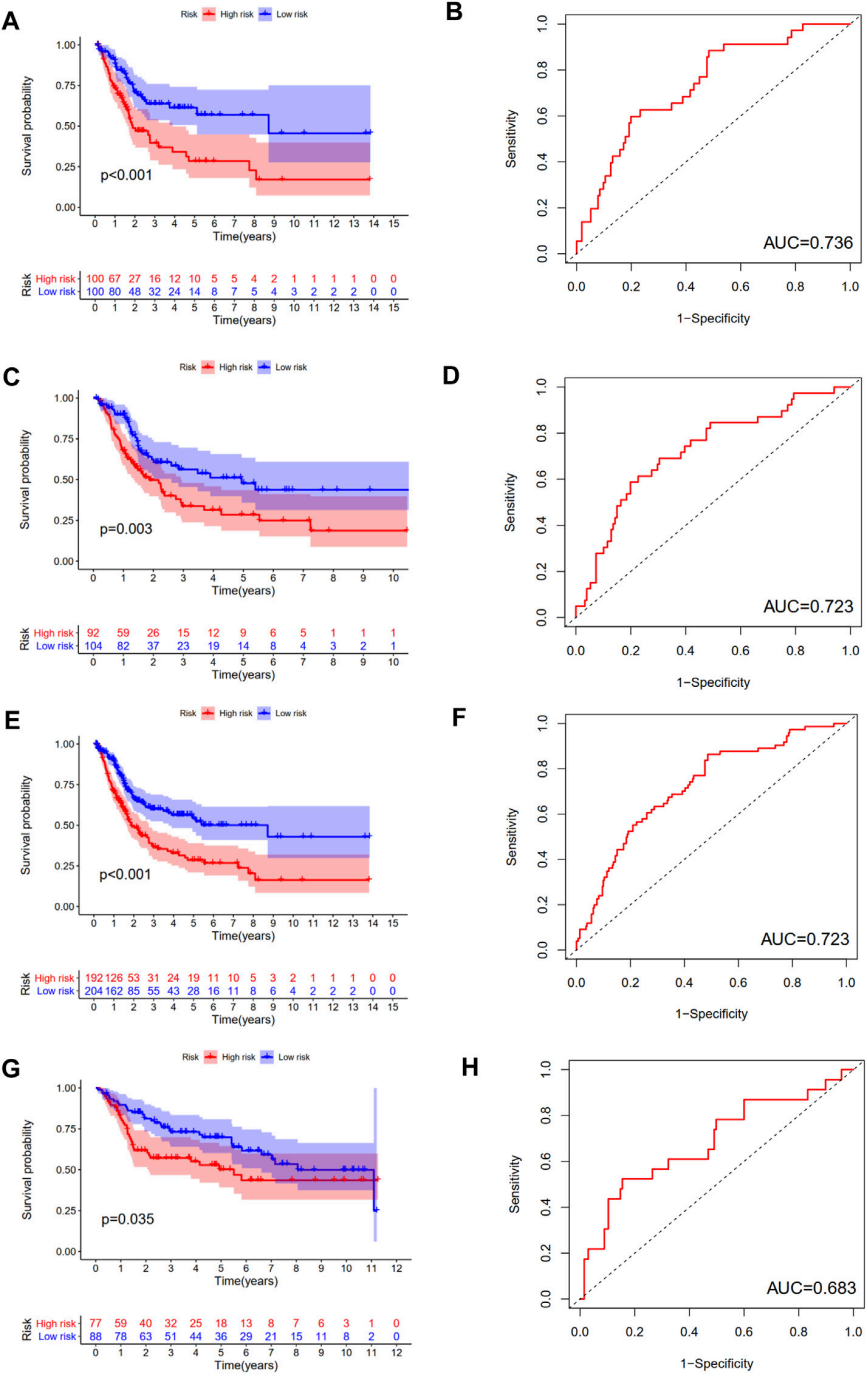
Internal and external verification of a novel ubiquitination-based prognostic index for BCa. The survival analysis and corresponding area under ROC curve in training cohort **(A**, **B)**, testing cohort **(C**, **D)**, the whole TCGA cohort **(E**, **F)**, and validating cohort **(G**, **H)**.

**FIGURE 6:**
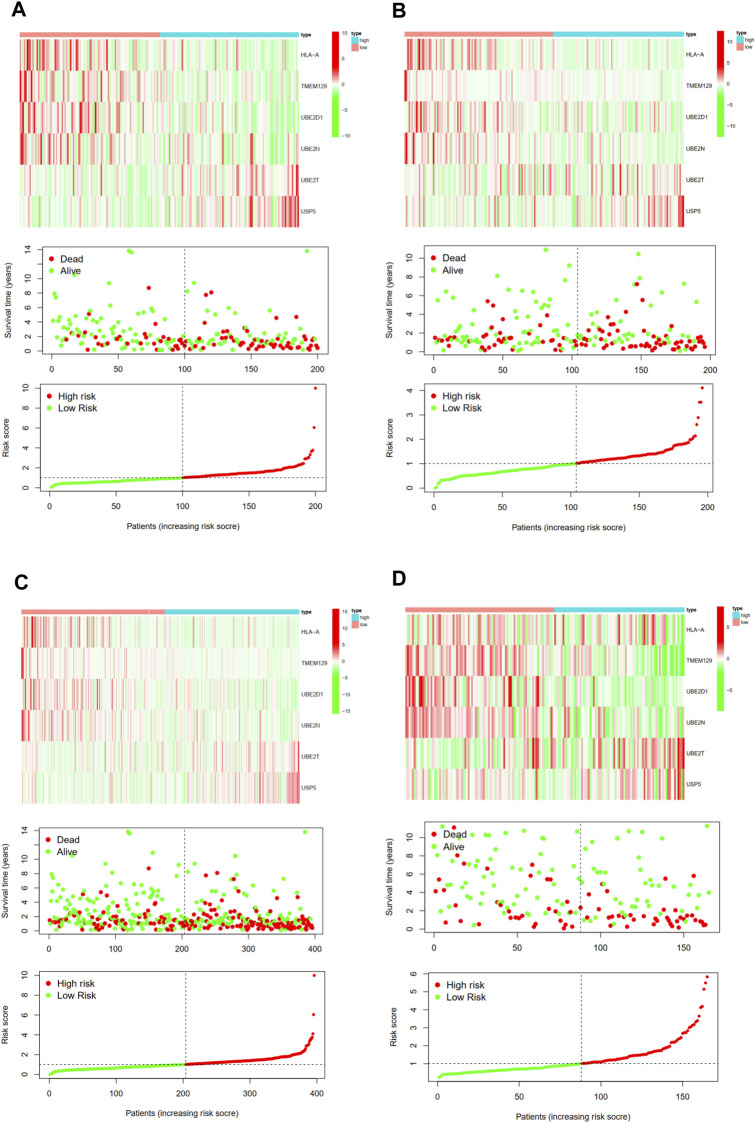
The expression heatmap, the distribution of risk score and survival time of training cohort **(A)**, testing cohort **(B)**, the whole TCGA cohort **(C)**, and validating cohort **(D)**.

Then, we calculated the risk score of each patient using above calculation formula in training cohort, testing cohort, and validating cohort. All patients were divided into high-risk score group and low-risk score group according to the median risk score. The difference in OS between low-risk and high-risk group was statistically significant in training cohort (*p* < 0.001), testing cohort (*p* = 0.003), whole TCGA cohort (*p* < 0.001) and validating cohort (*p* = 0.035), respectively. Low-risk score was associated with significantly preferable OS in comparison with high-risk score in training cohort, testing cohort, whole TCGA cohort and validating cohort, respectively. The area under ROC curve (AUC) for OS prediction was 0.736, 0.723, 0.723 and 0.683 in training cohort, testing cohort, whole TCGA cohort and validating cohort, respectively, suggesting the promising value of this novel ubiquitination-based prognostic index for prognosis prediction of BCa (Figure 5). The expression heatmaps, the distributions of risk score and survival time of training cohort, testing cohort, whole TCGA cohort and validating cohort were presented in Figure 6.

Univariate and multivariate independent prognostic analysis were used to demonstrate whether this index was an independent predictor of OS in BCa patients. Univariate analysis showed that age (*p* = 9.97E-05), stage (*p* = 9.56E-08), T stage (*p* = 6.08E-05), N stage (*p* = 1.57E-07) and this prognostic index (*p* = 1.96E-10) were associated with OS of BCa. Multivariate analysis demonstrated that only age (*p* = 0.000189) and this prognostic index (*p* = 3.82E-06) were independent predictors for OS of BCa, indicating the great performance of this prognostic index (Table 3).

**TABLE 3:** Univariate and multivariate independent prognostic analysis in whole TCGA cohort.

Id	Univariate				Multivariate			
	HR	Low 95% CI	High 95% CI	*p* Value	HR	Low 95% CI	High 95% CI	*p* Value
Age	1.034279	1.016869	1.051987	9.97E-05	1.033413	1.015734	1.051399	0.000189
Gender	0.87265	0.613469	1.241331	0.448681	-	-	-	-
Grade	4.369291	0.608939	31.35078	0.142481	-	-	-	-
Stage	1.86086	1.481237	2.337776	9.56E-08	1.298218	0.855003	1.971184	0.220644
T	1.656663	1.294413	2.120292	6.08E-05	1.268284	0.937045	1.716613	0.123823
N	1.564093	1.323311	1.848686	1.57E-07	1.196249	0.891274	1.605581	0.232718
Risk score	1.330154	1.21827	1.452313	1.96E-10	1.268876	1.146972	1.403736	3.82E-06

### Associations of this Prognostic Index with Clinicopathologic Features

**FIGURE 7:**
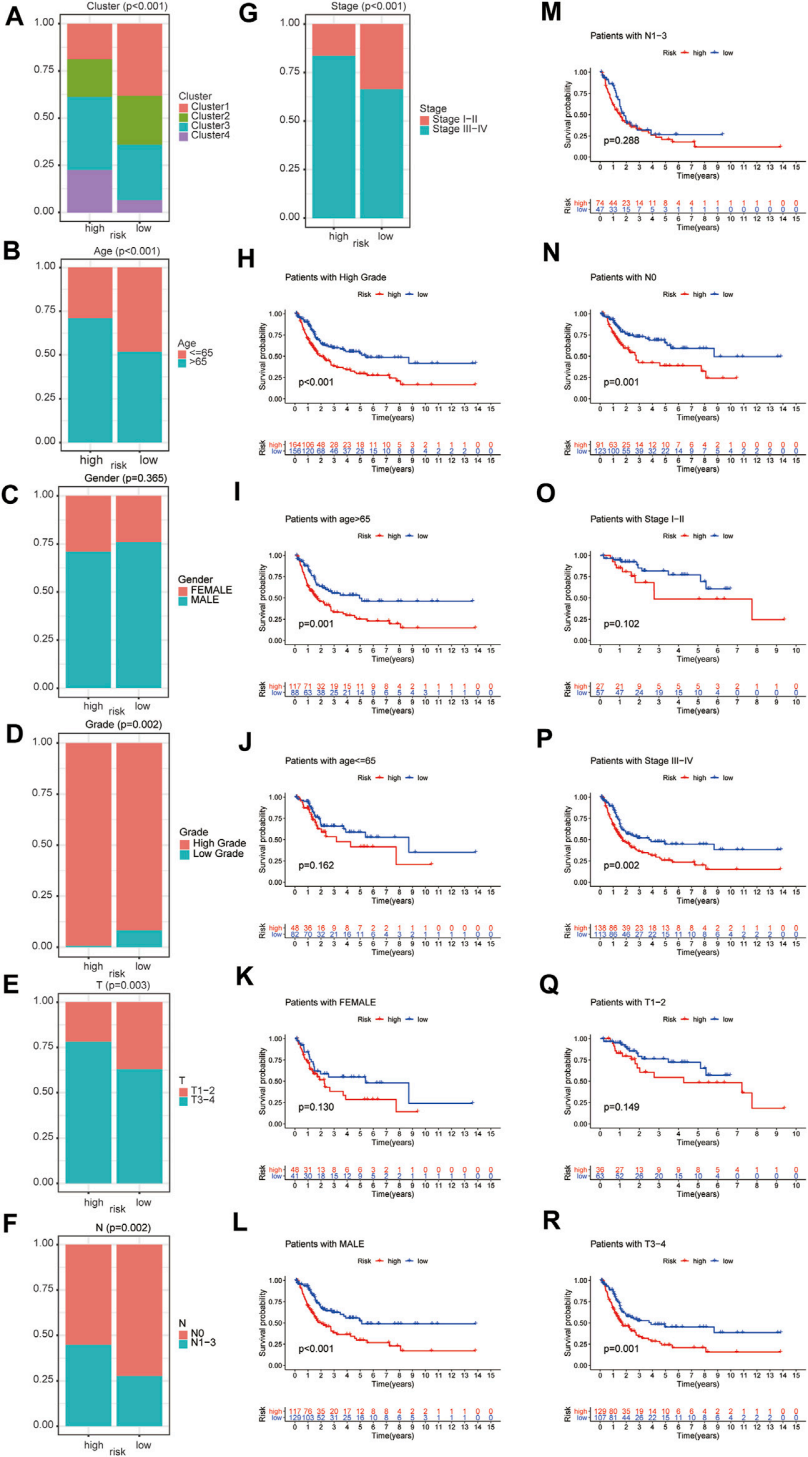
The difference of clinicopathologic features between high and low risk group, including ubiquitination-related molecular subtypes **(A)**, age **(B)**, gender **(C)**, grade **(D)**, T stage **(E)**, N stage **(F)**, and AJCC stage **(G)**. The survival analysis between high and low risk group in patients of high grade **(H)**, age ≤65 **(I)**, age >65 **(J)**, female **(K)**, male **(L)**, AJCC stage I-II **(M)**, AJCC stage III-IV **(N)**, T1-2 stage **(O)**, T3-4 stage **(P)**, N0 stage **(Q)** and N1-3 stage **(R)**.

The difference of clinicopathologic features between high and low risk group were explored. The results demonstrated that ubiquitination-related molecular subtypes (*p* < 0.001), age (*p* < 0.001), grade (*p* = 0.002), T stage (*p* = 0.003), N stage (*p* = 0.002), AJCC stage (*p* < 0.001) and were significantly different between high-risk and low-risk patients. However, gender (*p* = 0.365) was similar between high-risk and low-risk patients (Figures 7A–G).

### Subgroup Survival Analysis

Subgroup survival analysis demonstrated that this prognostic index was especially suitable for older, male, high grade, AJCC stage III-IV, stage N0, stage T3-4 BCa patients. Low-risk score was associated with significantly preferable OS in comparison with high-risk score in patients of age >65 or male or high grade or AJCC stage III-IV or N0 stage or T3-4 stage. However, the difference in OS between low-risk and high-risk group was not statistically significant in patients of age ≤65 or female or AJCC stage I-II or N1-3 stage or T1-2 stage (Figures 7H–R). Besides, considering the extremely low case number of patients with low grade, we did not conduct subgroup survival analysis in this subgroup.

**FIGURE 8:**
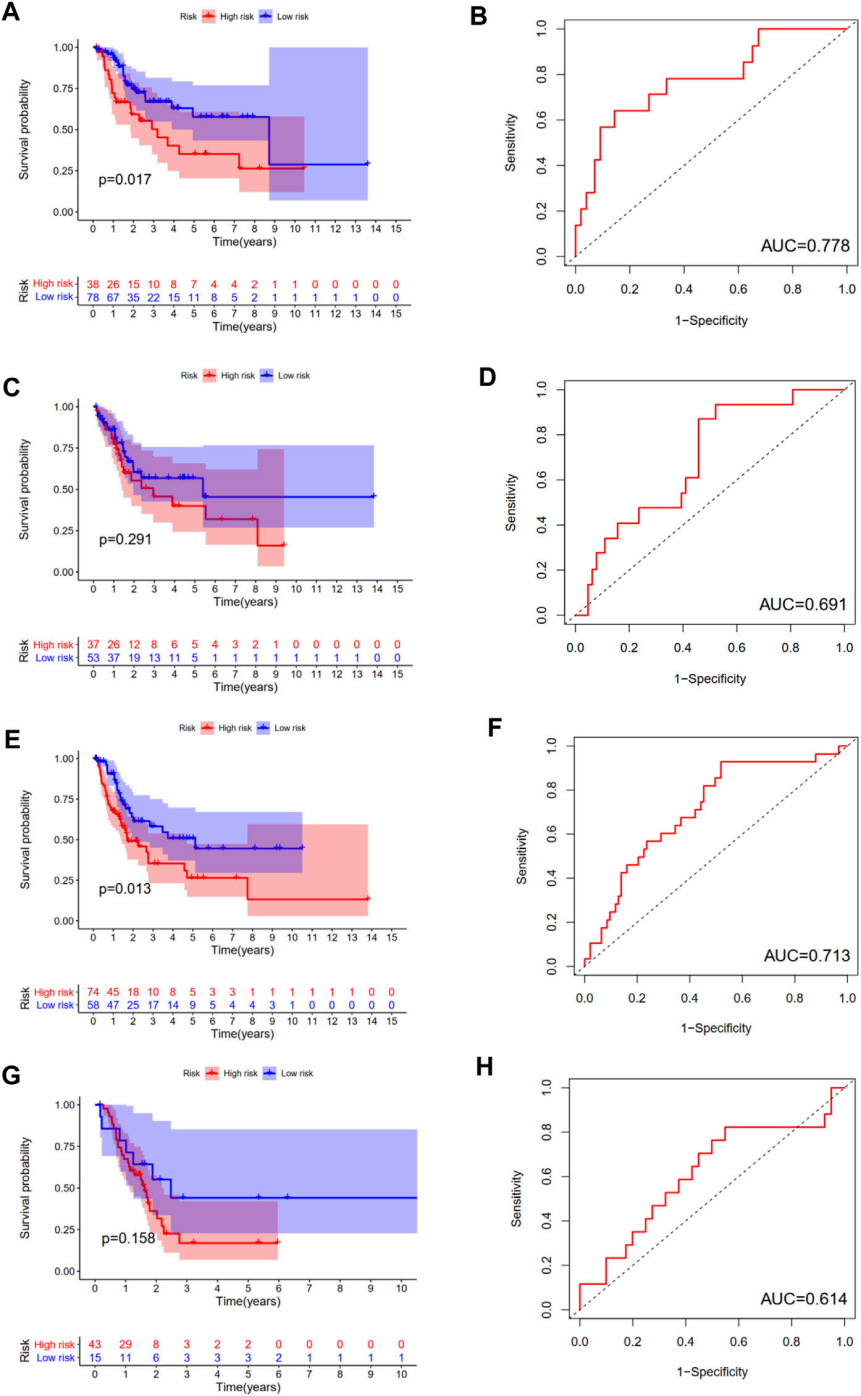
The survival analysis between high and low risk group, and corresponding area under ROC curve in BCa patients of subtype 1 **(A**, **B)**, subtype 2 **(C**, **D)**, subtype 3 **(E**, **F)**, and subtype 4 **(G**, **H)**.

We calculated the risk score for each patient of each ubiquitination-related molecular subtype. The results revealed that this prognostic index was especially applicable in BCa patients of subtype 1 and subtype 3. Low-risk score was associated with significantly preferable OS in comparison with high-risk score in BCa patients of subtype 1 and subtype 3. The area under ROC curve (AUC) for OS prediction was 0.778, 0.713 in BCa patients of subtype 1 and subtype 3, respectively. However, the difference in OS between low-risk and high-risk group was not statistically significant in subtype 2 and subtype 4 patients (Figure 8).

### Associations of this Prognostic Index with Immune Cell Infiltration and Tumor Microenvironment

**FIGURE 9:**
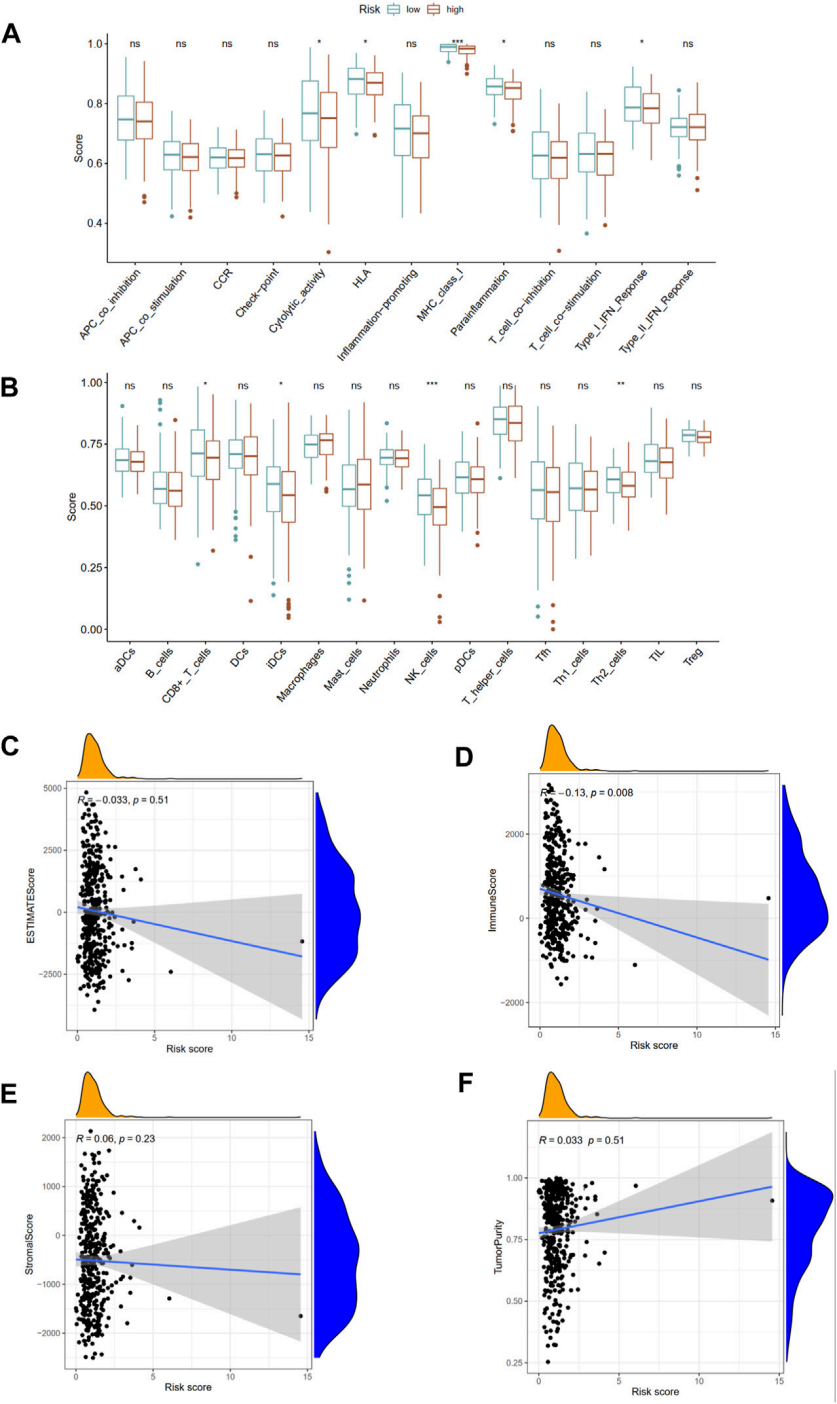
Associations of this prognostic index with immune functions **(A)** and immune infiltrating cells **(B)** in TCGA cohort. The association between risk score and ESTIMATE score **(C)**, immune score **(D)**, stromal score **(E)**, and tumor purity **(F)**. APC, antigen-presenting cells; CCR, chemokine receptors; HLA, human leukocyte antigen; MHC, major histocompatibility complex; IFN, interferon; DCs, dendritic cells; iDCs, interdigitating dendritic cells; pDCs, plasmacytoid dendritic cells; aDCs, activated dendritic cells; NK, natural killer; Th1, T helper 1; Th2, T helper 2. *p < 0.05; **p < 0.01; ***p < 0.001.

The immune functions and immune cells infiltration were quantified using ssGSEA in TCGA cohort. The infiltrating proportions of CD8+ T cells, interdigitating dendritic (iDC) cells, natural killer (NK) cells, T helper 2 (Th2) cells in low-risk group were significantly increased compared with that in high-risk group; patients with low-risk score have higher scores of immune function or pathways than those with high-risk, including cytolytic activity, human leukocyte antigen (HLA), major histocompatibility complex (MHC) class I, para-inflammation, type I interferon (IFN) response (Figures 9A,B). Besides, the associations of ubiquitination-based prognostic index with TME scores were explored. The correlation analysis demonstrated that the risk score was negatively associated with immune score derived from ESTIMATE algorithm. However, the risk score was not associated with ESTIMATE score, stromal score, and tumor purity score (Figures 9C–F).

### Validation of Six Risk URGs and GSEA Functional Enrichment

**FIGURE 10:**
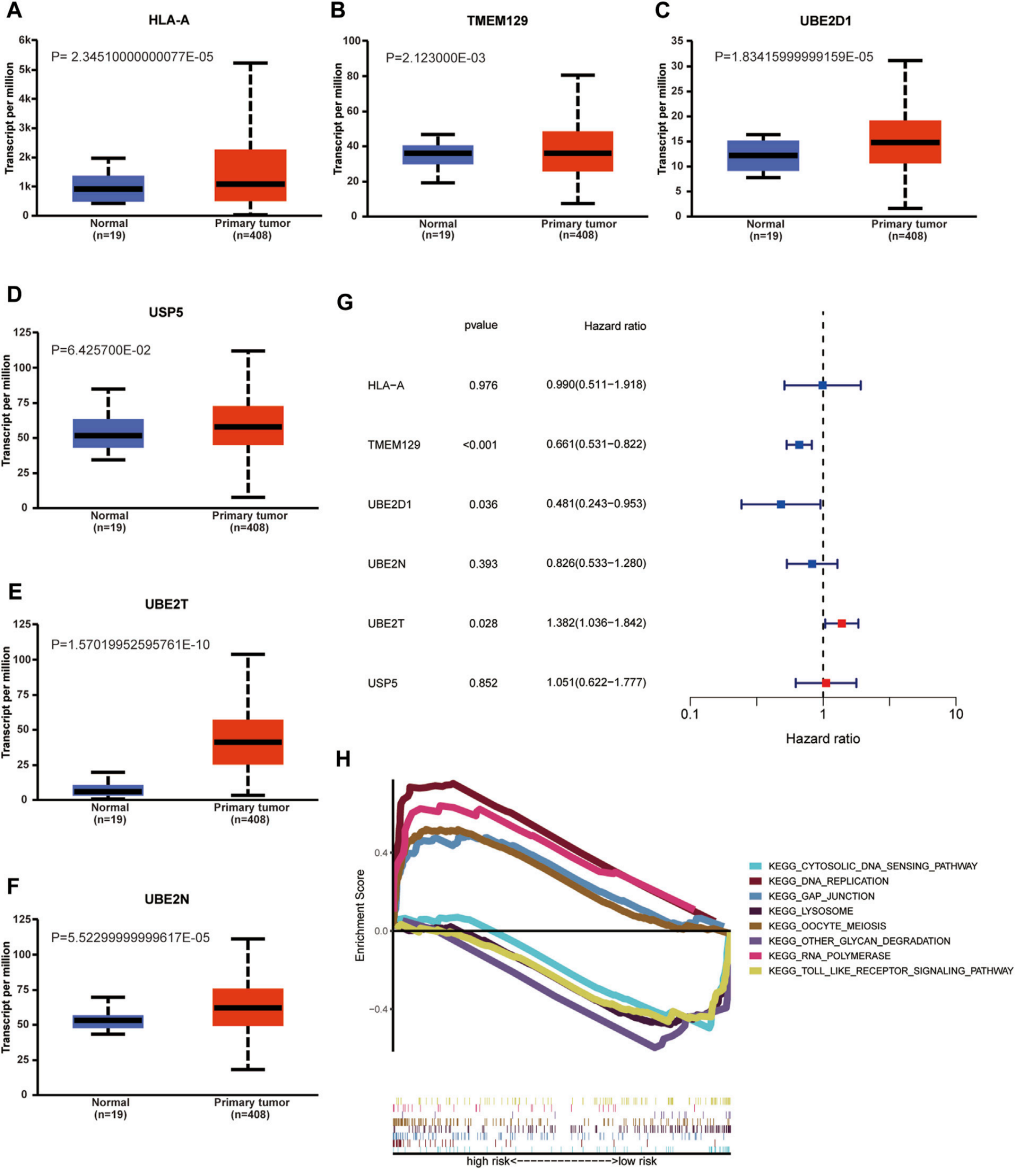
Validation of mRNA expression levels of HLA-A **(A)**, TMEM129 **(B)**, UBE2D1 **(C)**, USP5 **(F)**, UBE2T **(E)**, and UBE2N **(D)** in UALCAN database. Univariable Cox regression analysis in validating cohort (GEO cohort) to validate the prognostic value of risk URGs **(G)**. Kyoto Encyclopedia of Genes and Genomes (KEGG) functional enrichment of high-risk and low-risk group using Gene Set Enrichment Analysis (GSEA) method **(H)**.

As indicated by UALCAN database, the mRNA expression levels of HLA-A, TMEM129, UBE2D1, UBE2N, UBE2T in BCa tissue were significantly increased compared with that in normal tissue; however, the mRNA expression level of USP5 was similar between normal and tumor tissue. We performed univariable Cox regression analysis to validate the prognostic value of risk URGs in validating cohort (GEO cohort). The results showed that TMEM129, UBE2D1, and UBE2T were successfully validated to be associated with OS in GEO cohort. However, the prognostic value of HLA-A, UBE2T, and USP5 was not found to be associated with OS in GEO cohort. KEGG functional enrichment for high-risk group and low-risk group was performed using GSEA method. Toll like receptor signaling pathway, cytosolic DNA sensing pathway, other glycan degradation, and lysosome were major four KEGG enriched pathways in low-risk patients. GAP junction, DNA replication, oocyte meiosis, and RNA polymerase were major four KEGG enriched pathways in high-risk patients (Figure 10).

## Discussions

BCa is the ninth most common cancer worldwide and urothelial carcinoma accounts for 90% of all BCa cases [18–20]. Recently, it has been reported that the inducing accumulation of ubiquitinated proteins was considered as a vital treatment option for BCa [12]. Besides, immune checkpoint inhibitors have been confirmed significantly effective in treating BCa [21]. The identification of ubiquitination-related molecular subtypes and a novel prognostic index for BCa could classify the heterogeneous tumor microenvironment of BCa and contribute to the development of individualized therapy [22,23]. Previously, several gene expression based molecular classification systems with prognostic and predictive relevance have been described for MIBC. However, none of these classifications focused on ubiquitination [8,9].

In this study, we firstly identified a total of four molecular subtypes from the perspective of ubiquitination, which were different from previous studies. These four molecular subtypes were found to exhibit significantly diverse clinical characteristics and tumor microenvironment features. The difference of OS among these ubiquitination-related molecular subtypes was statistically significant. These four molecular subtypes had significantly different grade**.** The expression level of PD-L1 in subtype 3 and subtype 4 were significantly increased compared with that in subtype 1 and subtype 2. The activated mast cell was the exclusively discrepant immune infiltrating cell among these four ubiquitination-related subtypes. The ESTIMATE scores, immune scores, stromal scores in subtype 2 and subtype 3 were significantly higher in comparison with that in subtype 1 and subtype 4. Therefore, the four ubiquitination molecular subtypes would contribute to stratifying patients for PD-L1 expression level and prognosis. The underlying molecular mechanisms among the four ubiquitination molecular subtypes deserve further investigation.

Furthermore, we used multivariate Cox regression analysis to develop a novel ubiquitination-based index utilizing six URGs (including HLA-A, TMEM129, UBE2D1, UBE2N, UBE2T and USP5) for prognosis prediction of BCa. This novel ubiquitination-based prognostic index was successfully internally tested and externally validated. There were several previous studies developing prognostic index for BCa. Zhang et al. [24] included eight genes (AKAP12, ALDOB, CASP6, DTNA, HS3ST1, JUN, KDELR3, and STC1) to develop a novel hypoxia-related signature for BCa, which could play a precise role in predicting outcome. Liu et al. [25] used four prognostic genes (including ALOX5, FANCD2, HMGCR and FADS2) and multivariate Cox regression analysis to build a ferroptosis-related signature for BCa prognosis prediction. Qu et al. [26] developed a prognostic index for BCa from the perspective of immune-related gene. Luo et al. [27] constructed a poliovirus receptor (CD155)-related risk signature for predicting prognosis and immune infiltration of BCa. Du et al. [28] identified an epithelial to mesenchymal transition related lncRNA signature in order to predict BCa outcome. However, most of them did not conduct both internal and external verification, and this is the first study focusing on ubiquitination-related genes.

In this study, the difference in OS between low-risk and high-risk group was statistically significant in training cohort, testing cohort, and validating cohort, respectively. The AUC for OS prediction was 0.736, 0.723 and 0.683 in training cohort, testing cohort, and validating cohort, respectively, suggesting the promising value of this novel ubiquitination-based prognostic index for prognosis prediction of BCa. Subgroup analysis revealed that this prognostic index was especially suitable for older, male, high grade, AJCC stage III-IV, stage N0, stage T3-4 BCa patients. Moreover, we also found that this prognostic index was especially applicable in subtype 1 and subtype 3 BCa. Low-risk score was associated with significantly preferable OS in comparison with high-risk score in subtype 1 and subtype 3. The prognostic index could only be used to predict OS for subtype 1 and subtype 3 BCa.

The ubiquitination-related prognostic molecular index is composed of six risk URGs (including HLA-A, TMEM129, UBE2D1, UBE2N, UBE2T and USP5). Besides, In the identification of ubiquitination-related molecular subtypes, we revealed that there was a total of six URGs associated with OS, including CDC73, PRKDC, RNF40, TMEM129, UBE2N and USP5. Major histocompatibility complex, class I, A (HLA-A) belongs to the human leukocyte antigen (HLA) class I heavy chain paralogues [29]. Richard Golnik et al. [30] revealed that atypical ubiquitination is of great importance in modulating the processing of MHC class I antigen. Zhou et al. [31] demonstrated in their study that UBE2D1 was crucial in the development and progression of hepatocellular carcinoma. They also reported that high expression of UBE2D1 was attributed to the recurrent genomic copy number gain. However, no research paid attention to the oncogenic role of UBE2D1 in BCa. Jonine D Figueroa et al. [32] examined the data on a prevalent occupational exposure related to increased risk of BCa, and revealed a statistically significant additive interaction for rs798766 (TMEM129-TACC3-FGFR3). Yan et al. [33] demonstrated that miR-934 could bind to UBE2N mRNA and decrease the protein expression of UBE2N, and then lead to the cancer growth of BCa. Gong et al. [34] found that UBE2T might play momentous roles in BCa cells proliferation and cell cycle; hence, UBE2T might be a potential therapeutic targeting point for BCa. Xiao et al. [35] revealed that MAFG-AS1 could directly bind to HuR and then recruit USP5 to prevent HuR from degrading by de-ubiquitination. This process is vital in bladder urothelial carcinoma progression. Michael A Hahn et al. [36] reported that CDC73 is required for maintaining the mono-ubiquitination of H2B-K120 and the reduction of H2B-K120 mono-ubiquitination levels might be a potential mechanism of tumorigenic effect of CDC73. Shang et al. [37] demonstrated that DNA-PKcs (also called PRKDC) could negatively regulate the stability of cyclin B1 via ubiquitination. Fu et al. [38] summarized that E3 ligase RNF40 is vital in cancer development and metastasis. However, indeed, no research has been done yet in the aspect of these URGs in the progressions of BCa.

Toll-like receptors (TLRs) have been reported to drive immune responses in progression as well as treatment of cancer [39]. Recently, various TLRs signaling pathway proteins were proven to be related to the susceptibility of BCa [40]. TLRs agonists were regarded as effective immunostimulants with immunotherapeutic potential against BCa [41]. Besides, Bacillus Calmette-Guerin (BCG) was considered as an agonist of TLR2 and TLR4 and has been widely used in intravesical BCa therapy [41]. Connexins is of great importance for gap junctional intercellular communication and might facilitate the occurrence and metastases of cancer [42]. Ai et al. [43] revealed that GAP junction protein CX43 could be a promising therapeutic target for BCa. H B Grossman et al. [44] found that the alterations of connexin 26 expression may be associated with the malignant phenotype of BCa. In this study, we found that TLRs signaling pathway was a major enriched pathway in low-risk patients while the GAP junction pathway was a main enriched pathway in high-risk patients. However, further investigation into the specific mechanism is required in future.

There were several inescapable limitations of our study. Firstly, this study is retrospective and based on public database. The clinicopathologic information was not comprehensive and the detailed treatment regimens also could not be obtained. Validation using prospective sequencing data is required for generalizability. Secondly, these six risk URGs (including HLA-A, TMEM129, UBE2D1, UBE2N, UBE2T and USP5) might play vital roles in the development and progression of BCa. However, this study was performed via pure bioinformatics methods and the underlying mechanisms were not covered. Further *in vitro* and *in vivo* experiments into the underlying mechanism are required. Thirdly, it should be noted that for all parameters, the patient subgroup with the low case number showed statistically unsignificant differences of OS. Hence, one of the reasons for lack of significantly prognostic effect of the prognostic index in some patient subgroups could be the low case number. Validation of this ubiquitination-based prognostic index with larger sample size is required.

## Conclusion

In this study, we identified a total of four ubiquitination-related molecular subtypes with significantly different tumor microenvironment, prognosis, clinical characteristics and PD-L1 expression level. Besides, a novel ubiquitination-related prognostic index for BCa patients was developed and successfully verified. The ubiquitination-based molecular subtypes and prognostic index would contribute to further understanding of molecular mechanisms in BCa.

## Data Availability

Publicly available datasets were analyzed in this study. This data can be found here: TCGA database (https://portal.gdc.cancer.gov), GEO database (https://www.ncbi.nlm.nih.gov), Molecular Signatures Database (https://www.gsea-msigdb.org/gsea/msigdb) (MSigDB).
