# Supplementary material for: Ubiquitination-Related Molecular Subtypes and a Novel Prognostic Index for Bladder Cancer Patients
Source: Pathol Oncol Res. 2021 Oct 29;27:1609941. doi: 10.3389/pore.2021.1609941 (PMC8585742; doi:10.3389/pore.2021.1609941)
Supplement: Supplementary file 1 [file Table1.docx]

| **Supplementary Table 1. The list of ubiquitination-related genes (URGs)** | | | | |
| --- | --- | --- | --- | --- |
| Gene symbol | Official full name | Ensembl ID | Gene type |  |
| BCL10 | BCL10 immune signaling adaptor | [ENSG00000142867](http://www.ensembl.org/id/ENSG00000142867) | protein coding |  |
| CDC34 | cell division cycle 34, ubiqiutin conjugating enzyme | ENSG00000099804 | protein coding |  |
| CDC73 | cell division cycle 73 | [ENSG00000134371](http://www.ensembl.org/id/ENSG00000134371) | protein coding |  |
| CTR9 | CTR9 homolog, Paf1/RNA polymerase II complex component | [ENSG00000198730](http://www.ensembl.org/id/ENSG00000198730) | protein coding |  |
| DERL1 | derlin 1 | [ENSG00000136986](http://www.ensembl.org/id/ENSG00000136986) | protein coding |  |
| H2BC1 | H2B clustered histone 1 | [ENSG00000146047](http://www.ensembl.org/id/ENSG00000146047) | protein coding |  |
| H2BC10 | H2B clustered histone 10 | [ENSG00000278588](http://www.ensembl.org/id/ENSG00000278588) | protein coding |  |
| H2BC11 | H2B clustered histone 11 | [ENSG00000124635](http://www.ensembl.org/id/ENSG00000124635) | protein coding |  |
| H2BC12 | H2B clustered histone 12 | [ENSG00000197903](http://www.ensembl.org/id/ENSG00000197903) | protein coding |  |
| H2BC13 | H2B clustered histone 13 | [ENSG00000185130](http://www.ensembl.org/id/ENSG00000185130) | protein coding |  |
| H2BC14 | H2B clustered histone 14 | [ENSG00000273703](http://www.ensembl.org/id/ENSG00000273703) | protein coding |  |
| H2BC15 | H2B clustered histone 15 | [ENSG00000233822](http://www.ensembl.org/id/ENSG00000233822) | protein coding |  |
| H2BC17 | H2B clustered histone 17 | [ENSG00000274641](http://www.ensembl.org/id/ENSG00000274641) | protein coding |  |
| H2BC3 | H2B clustered histone 3 | [ENSG00000276410](http://www.ensembl.org/id/ENSG00000276410) | protein coding |  |
| H2BC4 | H2B clustered histone 4 | [ENSG00000180596](http://www.ensembl.org/id/ENSG00000180596) | protein coding |  |
| H2BC5 | H2B clustered histone 5 | [ENSG00000158373](http://www.ensembl.org/id/ENSG00000158373) | protein coding |  |
| H2BC6 | H2B clustered histone 6 | [ENSG00000274290](http://www.ensembl.org/id/ENSG00000274290) | protein coding |  |
| H2BC7 | H2B clustered histone 7 | [ENSG00000277224](http://www.ensembl.org/id/ENSG00000277224) | protein coding |  |
| H2BC8 | H2B clustered histone 8 | [ENSG00000273802](http://www.ensembl.org/id/ENSG00000273802) | protein coding |  |
| H2BC9 | H2B clustered histone 9 | [ENSG00000275713](http://www.ensembl.org/id/ENSG00000275713) | protein coding |  |
| HLA-A | major histocompatibility complex, class I, A | [ENSG00000206503](http://www.ensembl.org/id/ENSG00000206503) | protein coding |  |
| HLTF | helicase like transcription factor | [ENSG00000071794](http://www.ensembl.org/id/ENSG00000071794) | protein coding |  |
| LEO1 | LEO1 homolog, Paf1/RNA polymerase II complex component | [ENSG00000166477](http://www.ensembl.org/id/ENSG00000166477) | protein coding |  |
| OTULIN | OTU deubiquitinase with linear linkage specificity | [ENSG00000154124](http://www.ensembl.org/id/ENSG00000154124) | protein coding |  |
| PAF1 | PAF1 homolog, Paf1/RNA polymerase II complex component | [ENSG00000006712](http://www.ensembl.org/id/ENSG00000006712) | protein coding |  |
| PCNA | proliferating cell nuclear antigen | [ENSG00000132646](http://www.ensembl.org/id/ENSG00000132646) | protein coding |  |
| PEX10 | peroxisomal biogenesis factor 10 | [ENSG00000157911](http://www.ensembl.org/id/ENSG00000157911) | protein coding |  |
| PEX12 | peroxisomal biogenesis factor 12 | [ENSG00000108733](http://www.ensembl.org/id/ENSG00000108733) | protein coding |  |
| PEX13 | peroxisomal biogenesis factor 13 | [ENSG00000162928](http://www.ensembl.org/id/ENSG00000162928) | protein coding |  |
| PEX14 | peroxisomal biogenesis factor 14 | [ENSG00000142655](http://www.ensembl.org/id/ENSG00000142655) | protein coding |  |
| PEX2 | peroxisomal biogenesis factor 2 | [ENSG00000164751](http://www.ensembl.org/id/ENSG00000164751) | protein coding |  |
| PEX5 | peroxisomal biogenesis factor 5 | [ENSG00000139197](http://www.ensembl.org/id/ENSG00000139197) | protein coding |  |
| PRKDC | protein kinase, DNA-activated, catalytic subunit | [ENSG00000253729](http://www.ensembl.org/id/ENSG00000253729) | protein coding |  |
| RAD18 | RAD18 E3 ubiquitin protein ligase | [ENSG00000070950](http://www.ensembl.org/id/ENSG00000070950) | protein coding |  |
| RNF144A | ring finger protein 144A | [ENSG00000151692](http://www.ensembl.org/id/ENSG00000151692) | protein coding |  |
| RNF152 | ring finger protein 152 | [ENSG00000176641](http://www.ensembl.org/id/ENSG00000176641) | protein coding |  |
| RNF181 | ring finger protein 181 | [ENSG00000168894](http://www.ensembl.org/id/ENSG00000168894) | protein coding |  |
| RNF20 | ring finger protein 20 | [ENSG00000155827](http://www.ensembl.org/id/ENSG00000155827) | protein coding |  |
| RNF40 | ring finger protein 40 | [ENSG00000103549](http://www.ensembl.org/id/ENSG00000103549) | protein coding |  |
| RPS27A | ribosomal protein S27a | [ENSG00000143947](http://www.ensembl.org/id/ENSG00000143947) | protein coding |  |
| RRAGA | Ras related GTP binding A | [ENSG00000155876](http://www.ensembl.org/id/ENSG00000155876) | protein coding |  |
| RTF1 | RTF1 homolog, Paf1/RNA polymerase II complex component | [ENSG00000137815](http://www.ensembl.org/id/ENSG00000137815) | protein coding |  |
| SELENOS | selenoprotein S | [ENSG00000131871](http://www.ensembl.org/id/ENSG00000131871) | protein coding |  |
| SHPRH | SNF2 histone linker PHD RING helicase | [ENSG00000146414](http://www.ensembl.org/id/ENSG00000146414) | protein coding |  |
| TMEM129 | transmembrane protein 129, E3 ubiquitin ligase | [ENSG00000168936](http://www.ensembl.org/id/ENSG00000168936) | protein coding |  |
| UBA1 | ubiquitin like modifier activating enzyme 1 | [ENSG00000130985](http://www.ensembl.org/id/ENSG00000130985) | protein coding |  |
| UBA52 | ubiquitin A-52 residue ribosomal protein fusion product 1 | [ENSG00000221983](http://www.ensembl.org/id/ENSG00000221983) | protein coding |  |
| UBA6 | ubiquitin like modifier activating enzyme 6 | [ENSG00000033178](http://www.ensembl.org/id/ENSG00000033178) | protein coding |  |
| UBB | ubiquitin B | [ENSG00000170315](http://www.ensembl.org/id/ENSG00000170315) | protein coding |  |
| UBC | ubiquitin C | [ENSG00000150991](http://www.ensembl.org/id/ENSG00000150991) | protein coding |  |
| UBE2A | ubiquitin conjugating enzyme E2 A | [ENSG00000077721](http://www.ensembl.org/id/ENSG00000077721) | protein coding |  |
| UBE2B | ubiquitin conjugating enzyme E2 B | [ENSG00000119048](http://www.ensembl.org/id/ENSG00000119048) | protein coding |  |
| UBE2C | ubiquitin conjugating enzyme E2 C | [ENSG00000175063](http://www.ensembl.org/id/ENSG00000175063) | protein coding |  |
| UBE2D1 | ubiquitin conjugating enzyme E2 D1 | [ENSG00000072401](http://www.ensembl.org/id/ENSG00000072401) | protein coding |  |
| UBE2D2 | ubiquitin conjugating enzyme E2 D2 | [ENSG00000131508](http://www.ensembl.org/id/ENSG00000131508) | protein coding |  |
| UBE2D3 | ubiquitin conjugating enzyme E2 D3 | [ENSG00000109332](http://www.ensembl.org/id/ENSG00000109332) | protein coding |  |
| UBE2E1 | ubiquitin conjugating enzyme E2 E1 | [ENSG00000170142](http://www.ensembl.org/id/ENSG00000170142) | protein coding |  |
| UBE2E3 | ubiquitin conjugating enzyme E2 E3 | [ENSG00000170035](http://www.ensembl.org/id/ENSG00000170035) | protein coding |  |
| UBE2G1 | ubiquitin conjugating enzyme E2 G1 | [ENSG00000132388](http://www.ensembl.org/id/ENSG00000132388) | protein coding |  |
| UBE2G2 | ubiquitin conjugating enzyme E2 G2 | [ENSG00000184787](http://www.ensembl.org/id/ENSG00000184787) | protein coding |  |
| UBE2H | ubiquitin conjugating enzyme E2 H | [ENSG00000186591](http://www.ensembl.org/id/ENSG00000186591) | protein coding |  |
| UBE2J2 | ubiquitin conjugating enzyme E2 J2 | [ENSG00000160087](http://www.ensembl.org/id/ENSG00000160087) | protein coding |  |
| UBE2K | ubiquitin conjugating enzyme E2 K | [ENSG00000078140](http://www.ensembl.org/id/ENSG00000078140) | protein coding |  |
| UBE2L3 | ubiquitin conjugating enzyme E2 L3 | [ENSG00000185651](http://www.ensembl.org/id/ENSG00000185651) | protein coding |  |
| UBE2N | ubiquitin conjugating enzyme E2 N | [ENSG00000177889](http://www.ensembl.org/id/ENSG00000177889) | protein coding |  |
| UBE2Q2 | ubiquitin conjugating enzyme E2 Q2 | [ENSG00000140367](http://www.ensembl.org/id/ENSG00000140367) | protein coding |  |
| UBE2R2 | ubiquitin conjugating enzyme E2 R2 | [ENSG00000107341](http://www.ensembl.org/id/ENSG00000107341) | protein coding |  |
| UBE2S | ubiquitin conjugating enzyme E2 S | [ENSG00000108106](http://www.ensembl.org/id/ENSG00000108106) | protein coding |  |
| UBE2T | ubiquitin conjugating enzyme E2 T | [ENSG00000077152](http://www.ensembl.org/id/ENSG00000077152) | protein coding |  |
| UBE2V2 | ubiquitin conjugating enzyme E2 V2 | [ENSG00000169139](http://www.ensembl.org/id/ENSG00000169139) | protein coding |  |
| UBE2W | ubiquitin conjugating enzyme E2 W | [ENSG00000104343](http://www.ensembl.org/id/ENSG00000104343) | protein coding |  |
| UBE2Z | ubiquitin conjugating enzyme E2 Z | [ENSG00000159202](http://www.ensembl.org/id/ENSG00000159202) | protein coding |  |
| UCHL3 | ubiquitin C-terminal hydrolase L3 | [ENSG00000118939](http://www.ensembl.org/id/ENSG00000118939) | protein coding |  |
| USP5 | ubiquitin specific peptidase 5 | [ENSG00000111667](http://www.ensembl.org/id/ENSG00000111667) | protein coding |  |
| USP7 | ubiquitin specific peptidase 7 | [ENSG00000187555](http://www.ensembl.org/id/ENSG00000187555) | protein coding |  |
| USP9X | ubiquitin specific peptidase 9 X-linked | [ENSG00000124486](http://www.ensembl.org/id/ENSG00000124486) | protein coding |  |
| VCP | valosin containing protein | [ENSG00000165280](http://www.ensembl.org/id/ENSG00000165280) | protein coding |  |
| WAC | WW domain containing adaptor with coiled-coil | [ENSG00000095787](http://www.ensembl.org/id/ENSG00000095787) | protein coding |  |
| WDR61 | WD repeat domain 61 | [ENSG00000140395](http://www.ensembl.org/id/ENSG00000140395) | protein coding |  |
